# Computer-Aided Experiment Planning toward Causal Discovery in Neuroscience

**DOI:** 10.3389/fninf.2017.00012

**Published:** 2017-02-13

**Authors:** Nicholas J. Matiasz, Justin Wood, Wei Wang, Alcino J. Silva, William Hsu

**Affiliations:** ^1^Medical Imaging Informatics Group, Department of Radiological Sciences, University of California, Los AngelesLos Angeles, CA, USA; ^2^Silva Laboratory, Departments of Neurobiology, Psychiatry, and Psychology, Integrative Center for Learning and Memory, Brain Research Institute, University of California, Los AngelesLos Angeles, CA, USA; ^3^Department of Computer Science, Scalable Analytics Institute, University of California, Los AngelesLos Angeles, CA, USA

**Keywords:** epistemology, experiment planning, research map, causal graph, uncertainty quantification, information gain

## Abstract

Computers help neuroscientists to analyze experimental results by automating the application of statistics; however, computer-aided experiment planning is far less common, due to a lack of similar quantitative formalisms for systematically assessing evidence and uncertainty. While ontologies and other Semantic Web resources help neuroscientists to assimilate required domain knowledge, experiment planning requires not only ontological but also epistemological (e.g., methodological) information regarding how knowledge was obtained. Here, we outline how epistemological principles and graphical representations of causality can be used to formalize experiment planning toward causal discovery. We outline two complementary approaches to experiment planning: one that quantifies evidence per the principles of convergence and consistency, and another that quantifies uncertainty using logical representations of constraints on causal structure. These approaches operationalize experiment planning as the search for an experiment that either maximizes evidence or minimizes uncertainty. Despite work in laboratory automation, humans must still plan experiments and will likely continue to do so for some time. There is thus a great need for experiment-planning frameworks that are not only amenable to machine computation but also useful as aids in human reasoning.

## 1. Introduction

Much of the work in neuroscience involves planning experiments to identify causal mechanisms; however, neuroscientists do not use computers to plan future experiments as effectively as they use them to analyze past experiments. When neuroscientists perform experiments, analyze data, and report findings, they do much to ensure that their work is objective: they follow precise lab protocols so that their experiments are reproducible; they employ rigorous statistical methods to show that their findings are significant; and they submit their manuscripts for peer review to build consensus in their fields. In contrast, experiment planning is usually less formal. To plan experiments, neuroscientists find and read relevant literature, synthesize available evidence, and design experiments that would be most instructive, given what is known. Unfortunately, neuroscientists lack tools for systematically navigating and integrating a set of findings, and for objectively and exhaustively considering all causal explanations and experimental designs. Instead, when neuroscientists search for relevant information, they routinely rely on serendipity and their incomplete memory of publications. Similarly, when they synthesize evidence, neuroscientists often use unspecified methods, based mostly on implicit strategies that are accumulated through years of training. Although it applies to much of biology, this problem is particularly worrisome in neuroscience as researchers in this field often integrate information across multiple diverse disciplines, including molecular, cellular, systems, behavioral, and cognitive neuroscience. This methodological diversity and complexity could conceivably confound neuroscientists' search for the best experiments to perform next. The subjectivity of the experiment-planning process thus stands in stark contrast to the objectivity of the processes by which neuroscientists perform and analyze experiments.

This paper presents our perspective on computer-aided experiment planning and the role of graphical representations in formalizing causal discovery. After briefly describing ontologies and their role in experiment planning, Section 2 proposes that computer-aided experiment planning requires not only ontological but also epistemological information. After defining these two kinds of information, we outline how the latter can be used to formalize experiment planning. Section 3 discusses graphical representations of causality and their utility as formalisms for guiding experiment planning. First, essential features of causal graphs are introduced, including the concept of a *Markov equivalence class*. Next, we outline the components of a *research map*, a graphical representation of causality with epistemic elements relevant to experiment planning. Section 4 outlines how these two graphical representations of causality could be used to operationalize experiment planning toward causal discovery. Lastly, Section 5 speculates as to why a “statistics of experiment planning” has not been developed, and offers perspectives on the importance of a formal calculus of evidence for the future of scientific investigation.

## 2. Computer-aided experiment planning

Currently, ontologies and Semantic Web technologies help neuroscientists plan experiments by summarizing domain knowledge from the vast literature into forms more readily assimilated by individual researchers (Smith et al., 2007; Rubin et al., 2008; Fung and Bodenreider, 2012; Chen et al., 2013; Dumontier et al., 2013). Such resources classify phenomena hierarchically and describe relations that exist between them. Ontologies' usage can be divided broadly into three categories: knowledge management, data integration, and decision support (Bodenreider, 2008). Example applications include aiding drug discovery (Vázquez-Naya et al., 2010), identifying patient cohorts (Fernández-Breis et al., 2013), and facilitating manual literature curation (Krallinger et al., 2012). Widely used ontologies include the Gene Ontology (GO) (Ashburner et al., 2000), the Unified Medical Language System (UMLS) (Bodenreider, 2004), and the Systematized Nomenclature of Medicine—Clinical Terms (SNOMED CT) (Donnelly, 2006). While obviously useful, such resources often lack elements that we propose are critical for both identifying meaningful gaps in knowledge and planning experiments to mitigate them.

If experiment planning is to be formalized, it seems its operationalization must involve not only ontological principles but also epistemological ones. While ontological information tells us *what* exists, including objects, properties, and their relations, epistemological information entails descriptions of *how* we obtain this information. An epistemic statement can qualify an ontological assertion by describing both its truth value (e.g., the confidence attributed to knowledge) and its basis (e.g., the evidence that supports it) (de Waard and Maat, 2012). Epistemological methods for experiment planning would thus allow for the ranking of potential experiments, analogous to a cost function that directs the optimization of a model. The Ontology for Biomedical Investigations (Bandrowski et al., 2016) and the Evidence Ontology (Chibucos et al., 2014) are two recent knowledge bases that include epistemic elements (below, we outline quantitative approaches to such concepts). Knowledge-Engineering from Experimental Design (KEfED) is a formalism that captures both ontological and epistemological information by representing not only experimental findings but also semantic elements of the experiments themselves (Russ et al., 2011; Tallis et al., 2011). KEfED is based on the “Cycle of Scientific Investigation” (CoSI), a model in which (i) experiments induce observational and then interpretational assertions, and (ii) domain knowledge motivates hypotheses and then experimental designs (Russ et al., 2011). This paper addresses what we see to be a large asymmetry in this process: Scientists have robust statistical methods for validating observational assertions on the basis of experiments; however, scientists lack similar quantitative formalisms for justifying hypotheses on the basis of domain knowledge.

One way to operationalize experiment planning is thus to quantify the uncertainty of an existing model (or set of models); the goal of experiment planning is then to identify the experiment (or set of experiments) that would minimize uncertainty. A second complementary approach is to quantify experimental evidence; the goal of experiment planning is then to identify the experiment that maximizes evidence. (For interesting discussions of uncertainty and evidence, see Vieland, 2006; de Waard and Schneider, 2012, respectively.) Below, we outline these two strategies, which are meant to inform and complement the mostly implicit, creative processes currently used to plan experiments.

## 3. Graphical representations of causality

As a representation for ontological information (i.e., entities and their relations), graphical causal models (Spirtes et al., 2000; Koller and Friedman, 2009; Pearl, 2009) can been used as a tool for experiment planning (Pearl, 1995). Graphical models are a sensible formalism for guiding causal discovery: graphs concisely encode probabilistic relations between variables (Friedman, 2004); they are accessible to domain experts because they encode plain causal statements (as opposed to only statistical or probabilistic ones) (Pearl, 1995, 2009); and principled methods exist for assembling fragments of graphical models into one (Friedman, 2004; Cohen, 2015), a strategy that resembles the way researchers integrate facts from various sources. After reviewing key aspects of causal graphs below, we briefly introduce the concept of a research map, another graphical representation of causality that, in addition to ontological information, includes epistemological (specifically, methodological) information regarding the evidence behind causal assertions.

### 3.1. Causal graphs

A causal model can encode the causal structure of its variables with a *causal graph*. A causal graph is a directed graph with a set of variables (nodes) and a set of directed edges among the variables. A directed edge between two variables in the graph conveys that the variable at the tail of the edge has a direct causal effect on the variable at the head (Spirtes et al., 2000; Pearl, 2009).

Via its structure (i.e., its connectivity), a causal graph encodes probabilistic dependence and independence relations. The graphical criterion known as *d-separation* (Pearl, 2009) can be used to read such relations off a causal graph; d-separation thus translates the edges of a graph into probabilistic statements. There is a key connection between d-separation and probabilistic independence relations: considering a directed acyclic graph (DAG) with the causal Markov and causal faithfulness assumptions (Spirtes et al., 2000), any independence implied by d-separation holds if and only if the probability distribution associated with this DAG also exhibits this independence (Pearl, 2009).

#### 3.1.1. Markov equivalence classes

Per the rules of d-separation, even if two or more causal graphs have different structures, they can encode the same (in)dependencies. A set of causal graphs that all imply the same (in)dependencies is called a *Markov equivalence class* (Spirtes et al., 2000), or simply an equivalence class. The right-hand side of **Figure 2** gives an example of an equivalence class consisting of three unique graphs: *X* → *Y* → *Z*; *X* ← *Y* → *Z*; and *X* ← *Y* ← *Z*. Although the graphs disagree on the orientation of the edges, they all imply the same (in)dependence relations: *X* 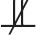 *Y*; *Y* 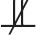 *Z*; *X* 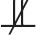 *Z*; and *X* ⫫ *Z* | *Y*. Thus, these graphs are observationally Markov equivalent—i.e., they are indistinguishable given only the observed (in)dependence relations.

It is important to note that an equivalence class can be extremely large; the number of possible causal graphs is super-exponential in the number of variables in the model. For a system with only six variables, there are over three million possible causal graphs (Robinson, 1973); if we allow for feedback (cyclicity), there are 2^30^ possible graphs. Causal discovery algorithms (that is, methods to identify the causal structure of a system) often cannot fully specify a single causal graph that accounts for the data; instead, they identify an equivalence class of graphs that satisfy the given (in)dependence relations. With only observational data, the graphs in an equivalence class will share the same adjacencies and vary in their edges' orientations. Interventional data, where the experimenter manipulates one of the variables, can eliminate specific causal structures from consideration.

### 3.2. Research maps

**Figure 1:**
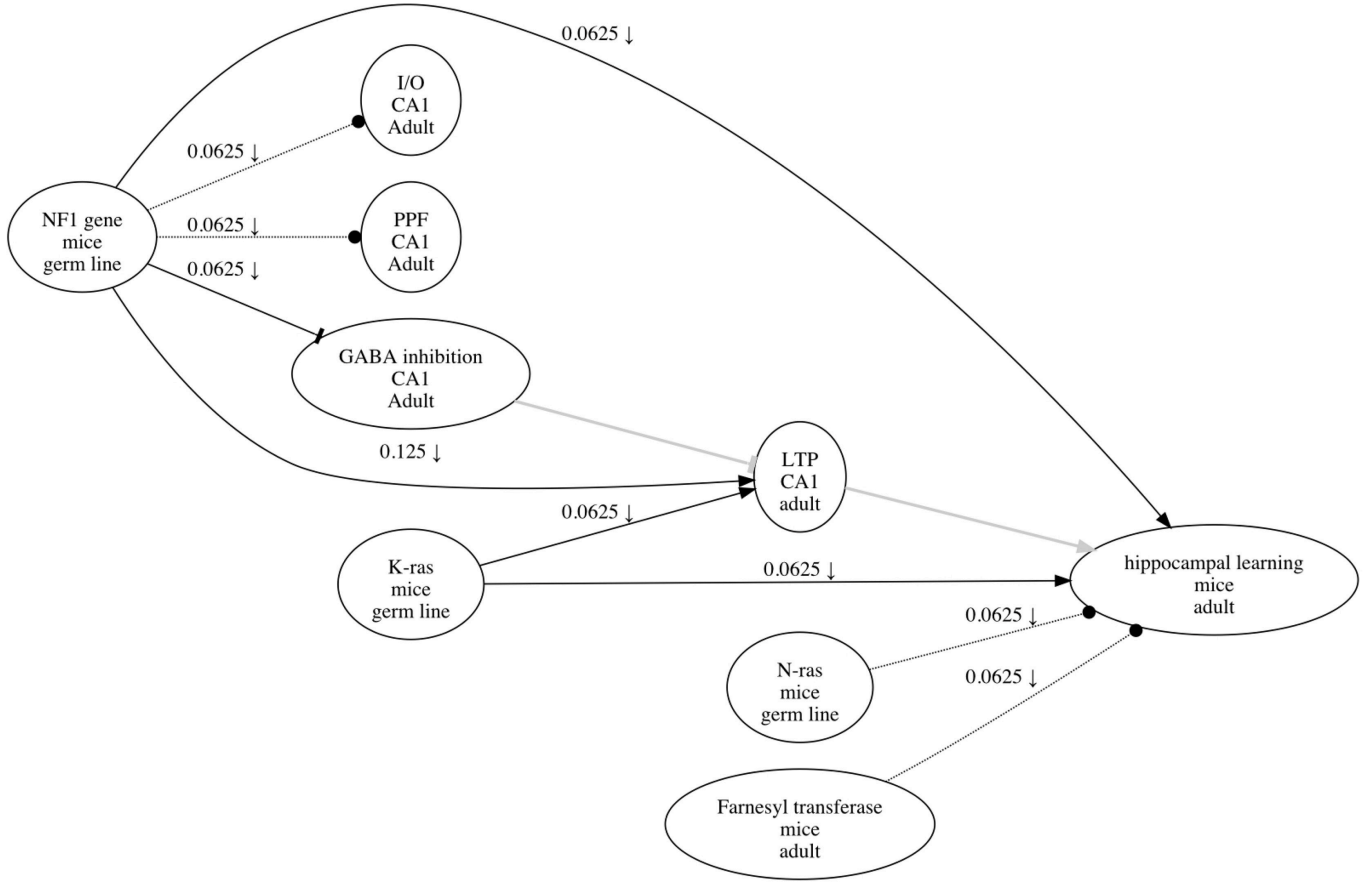
**An example of a research map that depicts the causal information in Costa et al. (2002)**. Each of the three types of causal relations are shown—for example, an excitatory edge from K-ras to LTP, an inhibitory edge from NF1 to GABA inhibition, and a no-connection edge from N-ras to hippocampal learning. The symbol on the edge from NF1 to hippocampal learning (↓) indicates that at least one negative intervention experiment was performed to test the relation between these two phenomena. The edges in gray (from GABA inhibition to LTP, and from LTP to hippocampal learning) are hypothetical edges: putative causal assertions for which the research article does not present empirical evidence. Hypothetical edges are useful for incorporating assumptions or background knowledge about a causal system; they give the research map additional structure to facilitate interpretation of the empirical results.

Although experimental data can be used to derive causal graphs, the source data from publications are often not available. Given the abundance of research articles, a lack of source data could be addressed in part by methods to extract causal information from literature. One such method is to annotate literature using a *research map*, a graphical representation with epistemic components relevant to experiment planning. Like a causal graph, a research map is a graphical representation of causal assertions, but it also includes methodological information pertaining to the evidence for these assertions. Such evidence is assessed using integration principles that operationalize experimental strategies for testing causal relations; these same principles can be used prospectively and explicitly to plan experiments (Landreth and Silva, 2013; Silva et al., 2014; Silva and Müller, 2015). See Figure 1 for an example of a research map, which was created using ResearchMaps^1^, a web application that implements this framework^2^.

Although, for usability, ResearchMaps implements a simple approach to identify nodes, the research map representation is agnostic to the specific ontology used, and can in principle identify nodes with any ontology. Research maps therefore should be generally applicable, as they instead emphasize the epistemological information used to gauge experimental evidence.

In a research map, each node represents a biological phenomenon (e.g., a protein, behavior, etc.), and each directed edge (from an *agent* node to a *target* node) represents one of three possible types of causal relations: (i) an excitatory edge (sharp arrowhead) indicates that the agent promotes its target; (ii) an inhibitory edge (flat arrowhead) indicates that the agent inhibits its target; and (iii) a no-connection edge (dotted line; circular arrowhead) indicates that the agent has no measurable effect on its target^3^. Because they represent phenomena and their relations, a research map's nodes and edges are thus ontological components.

To complement this ontological information, annotations on the edge of a research map give epistemological information regarding the type and amount of evidence for the edge's relation. The *type* of evidence for an edge is conveyed via symbols, one for each of four possible types of experiments that can give evidence for the relation. In a (i) positive intervention experiment (↑) and a (ii) negative intervention experiment (↓), either the quantity or probability of the agent is actively increased or decreased, respectively; in a (iii) positive non-intervention experiment (∅^↑^) and a (iv) negative non-intervention experiment (∅^↓^), an increase or decrease, respectively, in either the quantity or probability of the agent is observed, without intervention. The *amount* of evidence represented by an edge is conveyed by a score that, for convenience, ranges from zero to one. The score is calculated using integration principles (Silva et al., 2014) whose semantics reflect two common modes of reasoning in neuroscience: consistency and convergence. A detailed description of this score's calculation is beyond the scope of this article; we instead outline the epistemological concepts behind the score as they relate to experiment planning.

The integration principle of *consistency* states that evidence for a particular causal relation is stronger when an experiment is repeated and produces the same result. The consistency (i.e., the reproducibility) of a finding is important because any one experiment is always prone to errors and artifacts. Neuroscientists repeat experiments to mitigate this issue. The integration principle of *convergence* states that evidence for a particular causal relation is stronger when different types of experiments (e.g., positive and negative interventions and non-interventions) produce evidence for the same type of causal relation. The convergence of a finding is important because any one type of experiment, even when repeated multiple times with consistent results, can be biased and thus give a misleading perspective on the system under consideration. By performing multiple types of experiments, neuroscientists mitigate the risk of experimental artifacts.

## 4. Planning experiments with graphical representations of causality

Below we outline two complementary approaches to experiment planning using graphical representations of causality. In the first approach, a constraint-based algorithm is used to find the equivalence class that satisfies a set of causal-structure constraints (Hyttinen et al., 2014). Characterization of the equivalence class's uncertainty (underdetermination) then provides a quantitative framework for experiment planning, where the goal is to minimize uncertainty. In the second approach, the integration principles of research maps are extended to multiple edges to provide additional guidelines for experiment planning, where the goal is to maximize evidence.

### 4.1. Minimizing uncertainty in an equivalence class

Due to the enormity of the causal model space, a researcher is unlikely to be able to consider all of the causal graphs whose structures accommodate a set of results. What seems more likely is that domain knowledge and past experience will cause the researcher to subjectively prefer specific causal structures over others. Although this informed subjectivity can be practically useful, it could also bias researchers toward familiar causal structures. The method we outline below uses a computer to search the model space exhaustively; therefore, all graphs that accommodate either data or domain knowledge from the literature remain viable candidates. This approach has become feasible due to a recent advance in causal discovery (Hyttinen et al., 2014).

For this constraint-based approach to causal discovery, research maps can be used as an intuitive and accessible representation for neuroscientists to articulate causal-structure constraints in a familiar language (Silva et al., 2014). The edges in the resulting research maps can then be translated into constraints on causal structure, which are expressed probabilistically. For example, if an edge in a research map represents a positive intervention in an agent, *A*, and a resulting change in a target, *T*, this edge is translated into the causal-structure constraint *A* 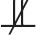 *T* | ∅ || *A*, which states that *A* is not independent of *T* when not conditioning on any variables, and when intervening on *A*.

**Figure 2:**
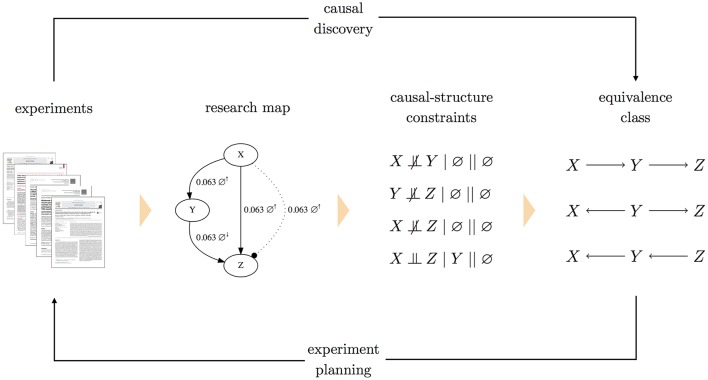
**A system diagram for planning experiments with causal graphs**. In this approach to experiment planning, research articles are annotated to produce a research map. Each edge in the research map is then translated into a causal-structure constraint of the form *A* ⫫ *B* | C || J, where C is a conditioning set and J is the intervention set. Both C and J can be the empty set (∅), as is the case for the non-intervention experiments depicted above (indicated by ∅^↑^ and ∅^↓^). To handle conflicting constraints, each causal-structure constraint is assigned a weight. A maximum-satisfiability solver then finds the causal graph that satisfies these constraints, while minimizing the sum of weights of (conflicting) unsatisfied constraints. With this one optimal graph, a forward inference method is used to identify the complete equivalence class of causal graphs that all imply the same (in)dependence relations. This equivalence class is then used as the basis for experiment planning. (Note that in the research map, the two experiments involving *X* and *Z* are shown as separate edges for clarity).

To accommodate cases of conflicting constraints, each constraint is assigned a weight, which represents a level of confidence. One option for weights is to use the scores of research map edges from which the constraints were derived. Epistemic information regarding the methodological diversity of how those constraints were derived would then inform the search over causal graphs. Assigning weights to constraints allows the causal discovery problem to be formulated as a constrained optimization: a Boolean maximum-satisfiability solver (Biere et al., 2009) searches for the causal graph that minimizes the sum of weights of unsatisfied constraints (Hyttinen et al., 2014). Having found the graph that is optimal in this sense, a forward inference method (Hyttinen et al., 2013) can be used to obtain the equivalence class of graphs that encode the same (in)dependence relations. A system diagram for this method is shown in Figure 2.

The uncertainty (i.e., underdetermination) of the resulting equivalence class can then be characterized, and experiment planning can be formalized as the search for experiments that would most effectively reduce this uncertainty. This experiment-selection criterion requires a metric that can quantify an experiment's reduction of uncertainty. We thus need a metric to characterize the uncertainty of an equivalence class, so that the metrics for different equivalence classes (i.e., before and after a particular experiment) can be compared. Below we outline a few approaches to defining such metrics.

A naïve approach would be to quantify the uncertainty of an equivalence class by simply counting the number of graphs it contains. By this metric, an equivalence class with *n* graphs would carry half the uncertainty of an equivalence class with 2*n* graphs. While perhaps useful, this metric fails to account for network connectivity (e.g., the existence and orientation of edges).

A more nuanced approach is the following “degrees-of-freedom” strategy. Consider that in any causal graph, each pair of variables will have one of four possible edge relations: (i) a “left-to-right” orientation (e.g., *X* → *Y*), (ii) a “right-to-left” orientation (e.g., *X* ← *Y*), (iii) neither orientation (e.g., *XY*),^4^ or, when we allow for feedback, (iv) both orientations (e.g., *X* ⇆ *Y*). Once a particular edge relation is instantiated for a pair of variables (e.g., *X* → *Y*), there are three other possible edge relations, three “degrees of freedom,” that the pair can take. For a system with *n* variables and thus (n2) pairs of variables, the number of degrees of freedom for a causal graph is then 3(n2). For each pair of variables in an equivalence class, we can count the number of instantiations that remain undetermined. For example, in the equivalence class of Figure 2, the graphs all agree that there is no edge between *X* and *Z* and that edges exist between the node pairs (*X, Y*) and (*Y, Z*); however, they specify different orientations for these latter two edges. By this metric, this equivalence class has two degrees of freedom. It may be useful to express this metric as a percentage: again, for the equivalence class in Figure 2, 2/(3(n2))≈22.2% of the degrees of freedom remain.

Yet a more nuanced approach is the following strategy, based on the concept of *edge entropy*. Tong and Koller (2001) consider graphs with three edge relations: *X* → *Y*, *X* ← *Y*, and *XY*. Given a distribution *P* over these relations, they quantify the uncertainty regarding the relation of an edge using the edge entropy expression

(1) $$\begin{array}{l} H(X,Y)=\;-P(X\to Y)\text{ log }P(X\to Y)\\ \;-P(X\leftarrow Y)\text{ log }P(X\leftarrow Y)\\ \;-P(X\;\;Y)\text{ log }P(X\;\;Y). \end{array}$$

This equation can be extended naturally to accommodate the fourth edge relation (i.e., *X* ⇆ *Y*) that was considered for the degrees-of-freedom metric:

(2) $$\begin{array}{l} H(X,Y)=\;-P(X\to Y)\text{ log }P(X\to Y)\\ \;-P(X\leftarrow Y)\text{ log }P(X\leftarrow Y)\\ \;-P(X\;\;Y)\text{ log }P(X\;\;Y)\\ \;-P(X⇆Y)\text{ log }P(X⇆Y). \end{array}$$

Instead of actual probabilities for this expression, we can use the empirical distribution exhibited by a given equivalence class, and include only terms with nonzero values for *P*(·). For example, in the equivalence class of Figure 2, one-third of the edges between *X* and *Y* show the causal parent as *X*, and two-thirds of the edges show the causal parent as *Y*. The entropy of this edge is then *H*(*X, Y*) = −(1/3) log (1/3) − (2/3) log (2/3) ≈ 0.918. For the edges between *Y* and *Z*, two-thirds show the causal parent as *Y*, and one-third shows the causal parent as *Z*. Similarly, the entropy of this edge is then *H*(*Y, Z*) = −(2/3) log (2/3) − (1/3) log (1/3) ≈ 0.918. Appropriately, once an edge's existence and orientation are determined, the entropy drops to zero. Such is the case for the edge relation between *X* and *Z* in the equivalence class of Figure 2: every graph agrees on the absence of this edge. The entropy of an entire equivalence class can then be defined as the sum (or average) of the entropies for every pair of variables in the system.

We can use such metrics to ask: Which experiment, if performed, would most effectively minimize the uncertainty of the equivalence class? The ideal experiment is then the one that minimizes these metrics. For example, when applied to the equivalence class of Figure 2, these metrics would prioritize experiments to test the relations between the pairs (*X, Y*) and (*Y, Z*): compared to the pair (*X, Z*), the other pairs have more degrees of freedom, and thus higher entropies. The possible outcomes of an experiment could be expressed in terms of the causal-structure constraints that could result, and these potential constraints could be used to determine the potential equivalence classes that could result from the experiment. The uncertainty metrics for both the current and prospective equivalence classes could be compared, yielding a method for quantifying the *information gain* of an experiment.

### 4.2. Maximizing evidence in a research map

Another approach to experiment planning is to rank experiments by the value of the evidence they could potentially yield. Given that convergence and consistency are used to gauge evidence in research maps, these principles can also be used to determine which experiments could most effectively strengthen or weaken the evidence for a particular edge. For example, if the evidence for an edge is based solely on a positive intervention experiment, then the principle of convergence would suggest that negative interventions and non-intervention experiments could be used to strengthen the evidence for that edge. Additionally, the principle of consistency would suggest that repetitions of any one of these experiments could strengthen the evidence. This reasoning represents a straightforward approach commonly used by neuroscientists to plan experiments. Beyond just single edges, these integration rules can be extended to entire research maps. To facilitate the presentation of these principles, we limit our discussion to research maps that contain only three nodes, representing part of a signal pathway or any other biological cascade.

It is important to remember that experiments are usually carried out with reference to a specific hypothesis that is commonly suggested by findings and theories. In research maps, hypotheses are represented by hypothetical edges. Unlike edges representing empirical experiments, hypothetical edges have no score or experiment symbols (see Figure 1). Hypothetical edges can thus organize and structure empirical edges based on actual experiments. Although the causal relations represented by hypothetical edges cannot always be directly tested—perhaps we lack the required tools—they nevertheless inform the choice among feasible experiments by contextualizing empirical results within specific theories, interpretations, etc.

With a given research map, we can use a number of principles, including the *pioneering* rule, to develop its evidence. This *pioneering* rule states that when a research map's edges imply the existence of an edge that spans other edges, testing this edge can significantly inform the model. For example, if we have a research map with empirical edges *X* → *Y* → *Z*, then designing an experiment to test the connection *X* → *Z* will likely be instructive as to whether *X* contributes to *Z*. Finding that manipulations of *X* reliably affect *Z*, for example, will provide further evidence for the existence of a pathway from *X* to *Z*.

Having considered all of the pairwise edges in a research map, we then refer to what we call the *weakest-link* rule. This rule simply states that edges with the lowest score (i.e., the least evidence) should receive the most attention when designing experiments to assess a given research map. Using the example above, if the *X* → *Y* edge has a score of 0.250 while the *Y* → *Z* edge has a score of 0.125, the weakest-link rule states that we should further test the *Y* → *Z* edge first. Note that once a particular edge has been selected for additional experiments, the single-edge integration rules of convergence and consistency (see Section 3.2) provide guidelines for selecting the optimal type of experiment to perform.

There are cases when the above rules cannot identify a single experiment that is optimal: there may be two or more experiment types (e.g., positive and negative interventions) that could (potentially) provide equally consistent and convergent evidence, given the experiments that have already been performed. In such cases we refer to what we call the rule of *multi-edge convergence*. This rule states that when given a choice between (potentially) equally convergent experiment types, we should select the type that is least represented of the experiments recorded for the entire research map. The rationale for this rule is that increasing the methodological diversity of a set of findings will lower the chances of systematic artifacts. For example, the prevalence of negative interventions depicted in Figure 1 would motivate the use of positive interventions, as well as non-interventions, to study this system.

These rules—(single-edge) consistency and convergence, the pioneering rule, the weakest-link rule, and multi-edge convergence—provide guidelines for experiment planning when working with research maps. These rules attempt to make explicit and quantitative the epistemological strategies commonly used by neuroscientists. In articulating and further extending these rules to larger networks, we are attempting to expand the research maps framework so that it is useful not only for representing results but also for planning experiments.

## 5. Discussion

In this paper, we outline ways to formalize experiment planning in the context of causal discovery. These methods are not designed to replace inspiration or creativity in science; for example, they cannot determine the topics that scientists should pursue. These methods could instead help scientists to quantify and communicate the rationale for selecting particular experiments when testing an hypothesis. Just as statistical methods convey the significance of a finding, we propose that formalisms are needed to make the experiment-planning process more objective—e.g., by quantitatively assessing the amount of information (i.e., the *information gain*) that could be gleaned from a particular set of experiments.

In the last few decades, the causal modeling community has developed robust formalisms and algorithms for representing and identifying causal relations. Despite these advances, these methods remain surprisingly underused by neuroscientists seeking to identify causal mechanisms. Part of this trend is likely due to a lack of communication between researchers in these fields: many neuroscientists simply lack fluency in these methods, and thus do not use them. However, even for neuroscientists who wish to leverage the robust methods that causal models afford, there are significant challenges when applying these methods, given the many practical constraints imposed.

The first experiment-planning approach we propose is our attempt to render these methods usable by practicing neuroscientists, such that literature, in addition to data, can be used to derive causal graphs. If such methods are adopted, experiment planning will be made more objective, systematic, and communicable to the research community: potential experiments could be selected on the basis of their ability to reduce the space of possible causal graphs. The second approach proposed is our attempt to express epistemological principles (already used in neuroscience) in a quantitative framework to guide experiment planning. Together, these approaches form the basis of a mathematical framework that could be used in the scientific method alongside statistics: quantitative formalisms would then be used not only to validate scientific findings but also to justify the experiments themselves.

## Author contributions

NM, AS, JW, and WH contributed to the development of the two experiment-planning approaches presented. NM and AS wrote the manuscript. WW provided valuable advice for the project, and helped to edit the manuscript.

## Funding

This work was supported by the Leslie Chair in Pioneering Brain Research to AS, an NIH T32 (5T32EB016640-02) to NM, and an NIH-NCI T32 (T32CA201160) to JW. This project also received support from the NIH/NCATS UCLA CTSI Grant Number (UL1TR000124).

### Conflict of interest statement

The authors declare that the research was conducted in the absence of any commercial or financial relationships that could be construed as a potential conflict of interest. The reviewer MB and handling Editor declared their shared affiliation, and the handling Editor states that the process nevertheless met the standards of a fair and objective review.

## Footnotes

^1^http://researchmaps.org/.

^2^ResearchMaps allows a user to create a research map for an article in a few steps: (i) each experiment in the article, its type, and the identities of its agent–target pair are recorded; (ii) notes are made on the methods used to observe (or manipulate) the agent and to measure the change (or lack thereof) in the target; (iii) the result measured in the target is recorded. Users can enter this information either for empirical edges (with supporting experimental evidence in the article) or for hypothetical edges (with support from expert opinion or elsewhere in the literature). While the annotation of more established experimental methods may be straightforward, the annotation of work on the cutting edge of a field may be more open to interpretation, much like the findings themselves.

^3^The semantics of an edge in a research map differ from the semantics of an edge in a causal graph (see Section 3). One key distinction is that an edge in a research map does not necessarily imply a direct causal connection: the relation that is represented may instead be *ancestral*.

^4^The blank space between the two variables is intentional; it is meant to call attention to the fact that the corresponding nodes in the graph lack any type of edge between them.
